# Supplementary material for: Pigment Production Improvement in Rhodotorula mucilaginosa AJB01 Using Design of Experiments
Source: Microorganisms. 2021 Feb 14;9(2):387. doi: 10.3390/microorganisms9020387 (PMC7918216; doi:10.3390/microorganisms9020387)
Supplement: Supplementary file 1 [file microorganisms-09-00387-s001.zip › Table S1.docx]

**Table S1.** Plate count of sampling matrices for isolation of pigmented yeast.

| **Sample** | **Matrix** | **Colony Forming Units (CFU/mL or CFU/g)** | | | |
| --- | --- | --- | --- | --- | --- |
|  |  | **10^-1^** | **10^-2^** | **10^-3^** | **Pigmented Yeast** |
| M00 | Seawater | >300 | 254 | 143 | Negative |
| M01 | Wastewater | >300 | >300 | 284 | Negative |
| M02 | Clinical Wastewater | >300 | >300 | 274 | Negative |
| M03 | Stream Water | >300 | 239 | 167 | Negative |
| M04 | Fish digestive tract | 278 | 138 | 67 | Negative |
| M05 | Crab | 201 | 115 | 48 | 2 |
| M06 | Food | 170 | 92 | 16 | 8 |
