# Supplementary figures and images for: Pigment Production Improvement in Rhodotorula mucilaginosa AJB01 Using Design of Experiments
Source: Microorganisms. 2021 Feb 14;9(2):387. doi: 10.3390/microorganisms9020387 (PMC7918216; doi:10.3390/microorganisms9020387)

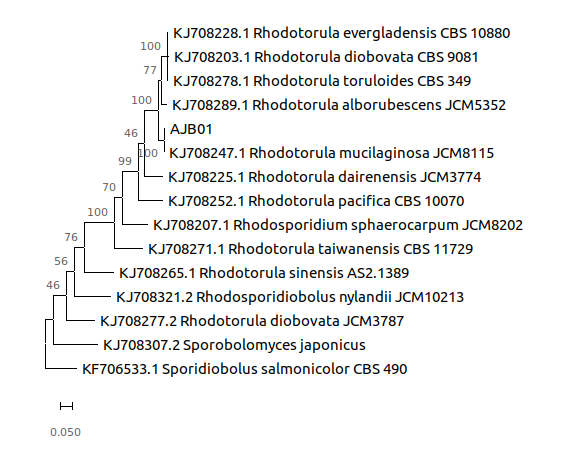

Supplement: Supplementary file 1 [file microorganisms-09-00387-s001.zip › Figure S1 feb 2021.tif]

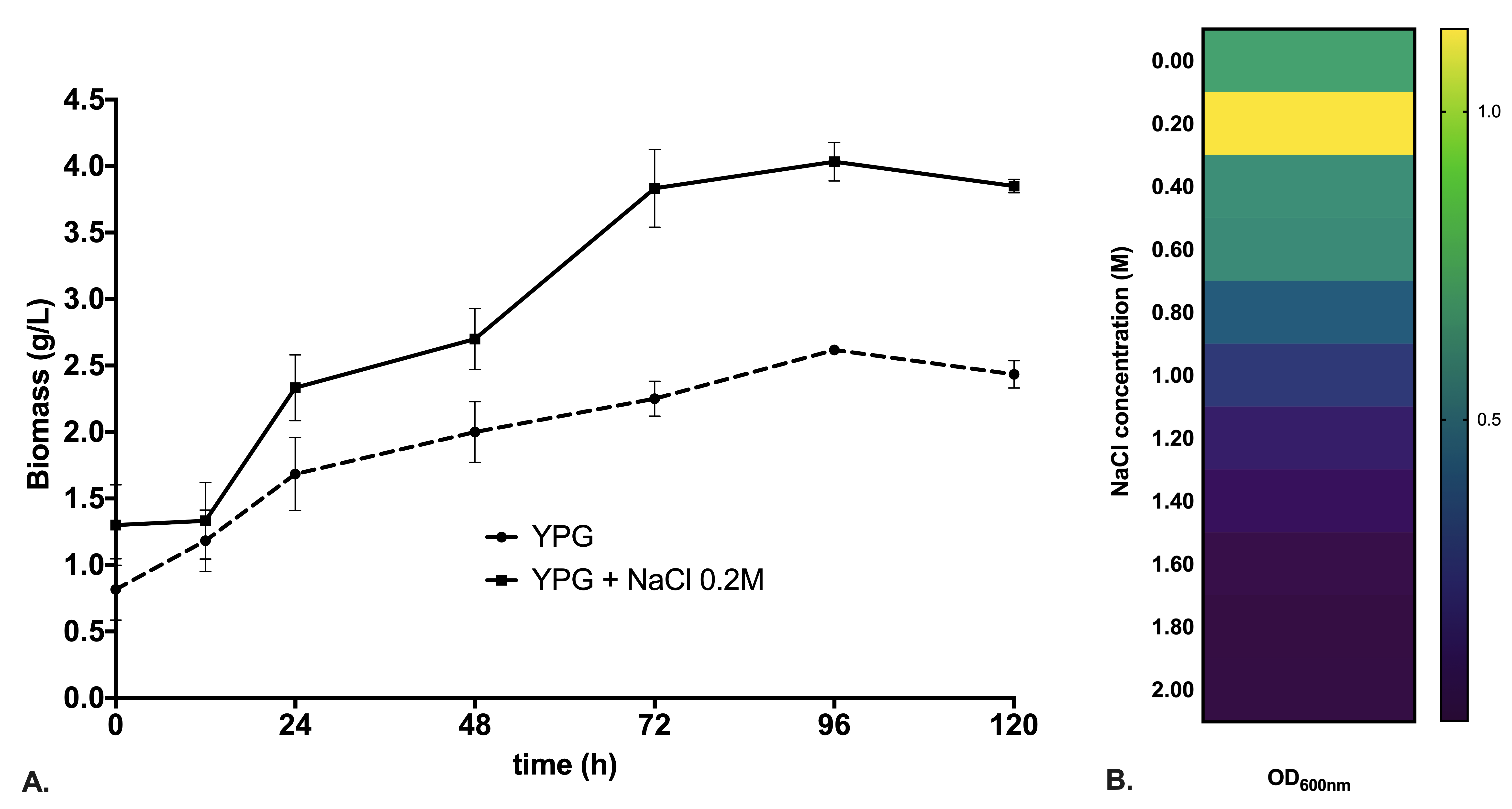

Supplement: Supplementary file 1 [file microorganisms-09-00387-s001.zip › Figure S2-feb-21.tiff]
